# Attitudes towards Tattoos among Spanish Health Science Students

**DOI:** 10.3390/ejihpe12120121

**Published:** 2022-11-22

**Authors:** Gemma Blázquez Abellán, Jesús López-Torres López, Mª José Moreno de la Rosa, Angel López González, Joseba Rabanales Sotos, Jesús Dativo López-Torres Hidalgo

**Affiliations:** 1 Department of Medical Sciences, Faculty of Pharmacy, University of Castilla-La Mancha, 02071 Albacete, Spain; 2 Group of Preventive Activities in the University Health Sciences Setting, University of Castilla-La Mancha, 02071 Albacete, Spain; 3 Department of Nursing, Physiotherapy and Occupational Therapy, Faculty of Nursing of Albacete, University of Castilla-La Mancha, 02071 Albacete, Spain; 4 Department of Medical Sciences, Faculty of Medicine, University of Castilla-La Mancha, 02071 Albacete, Spain

**Keywords:** attitudes, tattoo, university students, EAFT-D scale, health habits

## Abstract

(1) Background: The aim is to ascertain health science students’ attitudes towards tattoos and their association with healthy lifestyles and socio-demographic variables. (2) Methods: Descriptive study conducted on pharmacy, medical and nursing students (n = 423). To ascertain attitudes towards tattoos, we used the Attitudes Towards Tattoos Scale. Other variables were physical activity, healthy diet, harmful habits and socio-demographic variables. (3) Results: A total of 12.6% (95% CI 9.1–16.2) of students reported having a tattoo; 58.9% did not regard tattoos as a health risk. In terms of attitudes, the mean score in the range of 7–35 (7—most unfavourable to 35—most favourable) was 22.6 (SD 5.2; 95% CI: 22.0–23.2). Scores were higher (*p* < 0.05) among women (23.1; SD: 5.3), persons aged <20 years (23.6; SD: 5.0) and smokers (23.9; SD: 4.6). Attitudes were found to be more favourable (*p* < 0.05) in nursing students than in pharmacy or medical students. No relationship was observed with physical activity, healthy diet or drug use. (4) Conclusions: The attitude to tattoos is most favourable among women, persons aged under 20 years and nursing students. In terms of health habits, attitudes are more favourable among smokers, regardless of their level of physical activity, compliance with healthy eating guidelines or consumption of alcohol or other drugs.

## 1. Introduction

In recent years, there has been an increase, particularly among teenagers and young adults, in certain practices known as ‘body art’ or ‘body decoration art’ [1]. Tattooing is becoming an increasingly popular phenomenon in present-day society [2] and is acquiring steadily more interest from a scientific point of view due to its repercussions on health. A recent study undertaken in various countries across Europe, Asia and America [3] reported an overall prevalence of tattooed persons of 18.5%, with half having more than one tattoo. In 2018, Spain ranked sixth in the world in terms of the number of tattooed individuals. Despite the practice’s growth, a negative perception of tattoos continues to be held by a sector of the population, which considers that tattoos should be avoided in certain social and occupational spheres. In addition, it must be borne in mind that opinions about tattooed people can prove controversial, even in the health sector [4].

Among these techniques, tattooing is a body art that consists of creating an indelible mark, design or drawing on the skin. It is a body decoration procedure that, by means of puncture and micropigmentation techniques, brings about the rupture or perforation of the epidermal barrier through insertion into the skin of products containing dyes, pigments and auxiliary ingredients [5].

While the modification of physical appearance by tattooing has increased in frequency in western society in recent decades, the prevalence of these body modifications varies from one population to another and increases according to the age of the subjects studied [6,7]. This trend shows no signs of decreasing in the near future since, in recent years, adolescents and young adults have been getting increasingly more body tattoos, although the prevalence data cited in the medical literature are still limited [6,8]. It is estimated that over 100 million Europeans are tattooed [9]. In Germany, the prevalence of the practice of becoming tattooed is 8.5% in the general population, rising to a peak among the youngest subjects (age range 14–44 years). In Italy, other studies have observed a prevalence of 4.8% to 11.3% among secondary school students, while in the USA, the overall prevalence among university students has been reported as being 21.8% (23% men and 21% women) [8]. According to some authors, tattoo acquisition is currently similar in both sexes and includes adults and adolescents from different socio-economic groups and a wide range of occupations [10,11].

Body modifications have been a frequent phenomenon over the centuries, and though the current trend may seem new, it is a fact that these are very ancient practices employed by different cultures for a variety of purposes, ranging from aesthetic and religious to use as an identity mark or membership of a given group, tribe or family or as a sign of punishment or slavery. The scientific literature contains a great number of reasons why tattoos have currently acquired such popularity, e.g., the desire to celebrate a special occasion or relationship with a person, to feel unique or independent, to appear more attractive, to proclaim self-expression, to be creative, to express rebelliousness or an attraction to risk behaviours, to feel happier with/about oneself, etc. [12,13,14,15].

Young people feel attracted to body art and see it as a ‘different’ way of being themselves. However, one should not lose sight of the fact that it is an important socio-cultural phenomenon that is not free of health risks. Tattoos have numerous medical complications that have been described in various studies. Despite the new regulations designed to guarantee safe tattooing practices, tattoos are often done by unlicensed persons in unauthorised establishments [11], something that serves to increase health risks and give rise to diseases, both infectious (such as hepatitis B and C, HIV or tetanus) and non-infectious, with dermatitis and allergic reactions warranting special mention [11,16,17,18]. Similarly, there are studies that highlight the fact that tattoos may indicate problems of self-esteem or risk behaviour, including violence [19], drugs and alcohol [11,20,21,22,23], suicide [24], criminal behaviour patterns [25] and unprotected sexual activity [11] among adolescents in particular.

Currently, the increase in the popularity of tattoos and the influence of social networks on all students, including health science students, in regard to this topic is only too evident. Although health science students have been shown to be more knowledgeable about the health risks of tattoos [18], the influence of these social networks might nonetheless prove more important than university-based educational programmes. Even so, educational programmes can improve student knowledge about the health risks of tattoos and serve to prevent risk behaviours that might be associated with the practice [11]. Accordingly, the aim of this study is to ascertain health science students’ attitudes towards tattoos and their association with healthy lifestyles and socio-demographic variables.

## 2. Materials and Methods

### 2.1. Study Design and Participant Selection

We conducted a cross-sectional descriptive study on undergraduate pharmacy, medical and nursing students at the Albacete Biomedical and Health Sciences Campus (Bio-sanitary Campus). The study population consisted of students who were registered in each of the academic degree courses and voluntarily agreed to participate in the study by giving their oral informed consent, in all cases with the authorisation of the pertinent faculty management and teaching staff. The exclusion criterion was a refusal to participate in the study. The study protocol received official approval from the Clinical Research Ethics Committee of the Albacete University Teaching Hospital Complex (Spain) on 28 January 2020 (Nº 2019/10/095).

### 2.2. Study Variables

To evaluate attitudes towards tattoos in the university population, we used the Attitudes Towards Tattoos Scale (Escala de Actitudes frente al Tatuaje/EAFT-D), made up of 10 items representing pairs of adjectives and designed to obtain a general rating of tattoos in the following terms: positive–negative; agreeable–disagreeable; desirable–undesirable; ugly–attractive; delicate–aggressive; wrong–right; responsible–irresponsible; inappropriate–appropriate; conformist–rebellious; conventional–unconventional. This scale has a unifactorial structure, has shown high internal consistency, and its items possess a satisfactory discriminatory power [26]. Prior to use, the scale was adapted to Spanish by a translation/back-translation process in which two independent translators took part. Subsequently, the first version of the scale was obtained through a process of consensus between the research team and the two translators. Lastly, to assess the conceptual equivalence of the first Spanish version of the EAFT-D, a back-translation was made and compared against the original version, with any disagreements being settled by a new consensus.

In order to obtain the data, we designed a case report form that included the EAFT-D scale [26] along with other items pertaining to: tattoo acquisition (number, location, reasons, place of acquisition, information about health risks, etc.); level of physical activity as measured by the International Physical Activity Questionnaire (IPAQ) [27]; healthy eating criteria as per the Spanish Society for Community Nutrition (Sociedad Española de Nu-trición Comunitaria/SENC); alcohol consumption according to the Systematic Interview of Alcohol Consumption (Interrogatorio Sistematizado de Consumos Alcohólicos/ISCA questionnaire) [28]; and consumption of tobacco/other drugs and socio-demographic variables (age, sex, qualifications, size of town of origin, parents’ professions, and form of coexistence). This latter questionnaire was designed, revised and reviewed by experts in Preventive Medicine and Public Health.

### 2.3. Data Collection Procedure

The questionnaires were distributed to the students at the end of the academic year, with the exception of those who refused to participate or were not present at the time of its administration. The questionnaires were distributed in the lecture rooms and completed in a face-to-face setting. Each questionnaire was self-administered, and before answering it, the students were informed of the purpose of the study and the time required to complete the questionnaire, with the anonymity and confidentiality of their answers being assured. Stress was laid on the fact that participation was voluntary and no incentives were offered. The students were also informed that they could stop filling out the questionnaire at any time. During the administration of the questionnaire, a qualified person settled any queries or doubts raised and provided emotional support to students who needed it after completing the questionnaire. Students were informed of this before giving their consent.

### 2.4. Statistical Analysis

The participants’ responses were entered into a database, processed and subjected to statistical analysis. This consisted of a description of the study variables, including the construction of 95% confidence intervals, and the use of comparison of means tests (Student t, Mann–Whitney U and analysis of variance (ANOVA)), test of proportions (Chi-squared) and test of correlation analysis (Spearman correlation coefficient). A multiple linear regression model was then constructed to identify which of the variables had shown an association (*p* < 0.05) with attitudes towards tattoos in the bivariate analysis and maintained that association once the possible confounding factors (dependent variable: score obtained on the attitude scale) had been controlled for. The variables were introduced to the model by the stepwise method, and the coefficients were calculated using the least squares or maximum likelihood method. The independence of the residual values was checked by applying the Durbin–Watson test. All statistical analyses were performed using the IBM SPSS Statistics computer software programme version 19.0.

## 3. Results

Of a total of 423 students selected, 364 answered the questionnaire, thereby yielding an 86.1% response rate; the majority of responders were female (69.2%), with a mean age of 20.8 years (SD: 3.1). The remaining socio-demographic characteristics are shown in Table 1, with health habits in terms of the consumption of alcohol, tobacco and other drugs, diet and physical activity shown in Table 2.

A total of 12.6% (95% CI: 9.1–16.2) of all students reported bearing some type of tattoo, a figure corresponding to 8.9% of male students and 14.3% of female students, without this difference proving statistically significant. Among tattooed students, 48.9% bore more than one tattoo. Among those who had no tattoos, 45.7% indicated their intention to have one done. The majority (58.9%) did not regard the practice of tattooing as posing a health risk in terms of disease transmission; 19.9% of students considered that the practice posed no risk, while 45.8% considered that it posed very little risk.

When it came to the reasons for being tattooed, in 71.1% of cases, the students said that they had done so because of some personal experience (name of a loved one, important personal milestone, etc.). Other reasons given were: a sign of belonging to a group (sports team, music band, group of friends, etc.) or community in 11.1% of cases; aesthetic reasons (fashion or art) in 6.7% of cases; and no specific reason in 11.1% of cases.

Among those who had been tattooed, the preferred anatomical areas were the back, shoulder, legs and arms, with these students having gone to a specialised establishment to have their tattoos done in most cases (81.8%). The majority of tattooed students (93.3%) reported having received prior information about the risks linked to the practice of tattooing. After the completion of the tattoos, complications appeared in 8.9% of all tattooed students.

The proportion of tattooed subjects was significantly higher (*p* = 0.01) among first-year students (17.5%) versus the more senior students (8.9%) and higher (*p* < 0.001) among nursing (25.0%) versus pharmacy (9.2%) and medical students (9.6%). Similarly, the proportion of tattooed persons was higher (*p* < 0.001) among students who were smokers (28.2%) than among those who were non-smokers (8.9%) and higher (*p* = 0.004) among consumers of other drugs (35.3%) than among non-consumers (11.6%). Lastly, this proportion was likewise higher (*p* = 0.03) in students who came from towns with over 30,000 inhabitants (15.8%) than in those who came from towns with fewer inhabitants (8.0%).

Insofar as attitudes towards tattoos were concerned, it can be seen from Table 3 that on a score scale of 1 to 5, the highest proportion of responses corresponded to the value of 3 in each of the items or pairs of adjectives proposed, a value equidistant between favourable and unfavourable attitudes. Scale scores were calculated after excluding the pairs of adjectives ‘delicate–aggressive’, ‘conformist–rebellious’ and ‘conventional–unconventional’, which do not clearly express attitudes for or against. The mean score in a range of 7 to 35 (7—most unfavourable to 35—most favourable attitude) was 22.6 (SD: 5.2; 95% CI: 22.0–23.2).

Mean scores were significantly higher (*p* = 0.01) in female students (23.1; SD: 5.3) than in male students (21.6; SD: 4.7), with a difference in scores of 1.5 (95% CI: 0.3–2.7), and higher (*p* = 0.005) in persons aged under 20 years (23.6; SD: 5.0) than in older persons (22.0; SD: 5.2), with a difference in scores of 1.6 (95% CI: 0.5–2.7) and a weak, statistically significant (*p* = 0.003) negative correlation in evidence between the scores obtained and participants’ age (Spearman’s Rho = −0.161). Attitudes were also more favourable among smokers (23.9; SD: 4.6) than among non-smokers (22.3; SD: 5.3), with a difference in scores of 1.6 (95% CI: 0.2—3.0). In terms of academic degree courses, a more favourable attitude (*p* = 0.001) was found in nursing students (24.5; SD: 5.1) than in pharmacy (22.5; SD: 5.4) or medical students (21.5; SD: 4.6) and in first-year students (23.4; SD: 4.9) than in more senior students (22.0; SD: 5.3) (*p* = 0.009). No association was found between attitudes towards tattoos and level of physical activity, compliance with healthy eating guidelines, alcohol consumption or other harmful habits, or any of the remaining socio-demographic characteristics studied. Multiple linear regression showed that the most favourable attitudes were to be found among the youngest students, women, smokers, and nursing students (Table 4). While the regression equation’s explanatory capability was statistically significant (F = 5.140; *p* < 0.02), it nonetheless accounted for only 7.5% of the variability in attitudes towards tattoos. The Durbin–Watson test yielded a value of 1.753, thanks to which it was concluded that there was no correlation between the residual values.

## 4. Discussion

Attitudes towards tattoos are not at all uniform among students pursuing different degree courses and tend to be more favourable among the youngest students, who might be more easily influenced and have less health information. These attitudes could be very similar to those of other university students, not only because they are all equally young but also because it is a widely accepted social phenomenon. Similarly, attitudes are more favourable among women, who might be more concerned about body image, and among smokers, who already indulge in other behaviours that pose a risk to health. A number of authors have suggested that persons with body modifications are more prone to participate in risk behaviours or conduct [6,7,10,11], though there are still important gaps when it comes to whether healthy behaviours are inversely associated with body art techniques. Our study reflects that the proportion of tattooed persons was significantly higher among student smokers and users of other drugs, a situation previously described in women students and consumers of alcohol and marijuana [17,20,21].

The results of the study show that 12.6% of health science students have at least one tattoo, and among these, approximately half have more than one tattoo. In terms of sex, a higher number of females (14.3%) than males are tattooed (8.9%), without this difference being statistically significant. Other studies conducted on university students have yielded similar results, though with somewhat higher percentages [21]. As with our results, studies conducted in the USA show the prevalence of tattoos to be higher among women [11,23,29] and first-year students [12,13]. With respect to the higher number of tattoos found in the female population, it is contended [24] that women may be more prone to seek emotional solace and recovery by making greater use of this body art technique since a relationship between the presence of tattoos and levels of self-esteem and depression has been observed among women. It is also suggested that tattoos and other forms of body art, such as piercings, enjoy greater social acceptance among women, comparable to their perception of the use of cosmetics or jewellery [24]. As regards the intention of becoming tattooed among students who do not yet carry any tattoos, our results show that 45.7% would become tattooed in future. Earlier studies have reported this proportion to be 36.3% among Italian university students [17], with no differences between the sexes, and 30% among US university students [11].

When it comes to the reasons for having been tattooed, in most cases, our students reported having done so because of some personal experience (name of a loved one, important personal milestone, etc.). The reasons cited by other authors [6,8,10,11,17,21] as being most frequent are self-expression, a feeling of independence or aesthetic reasons, with other less common reasons being group pressure, rebelliousness if parents are not in agreement, or that it is fashionable. In contrast, among the reasons for not being tattooed, other authors [11] also cite aesthetic reasons, fear of pain or of contracting infections or a dislike of the effect that tattoos have on others.

As for the health risks posed by the practice of tattooing, in general, most students do not consider it a risk; in terms of disease transmission, this risk is regarded as very low, by almost half and even nil, by one out of every five responders. Other studies [8,10,11,17,25,29,30,31] have reported varying results in terms of the absence of health risks and the importance attributed to other problems such as skin irritations or other minor symptoms. These findings suggest that students who choose to be tattooed often do not take the risks into account and ignore any possible health consequences.

Furthermore, the practice of tattooing has been associated with other risk behaviours, such as violence [11]. Curiously, in one study conducted in Italy [6], a greater interest in tattoos was observed among science than arts students, albeit associated with unhealthy lifestyles, such as the consumption of harmful substances, including alcohol and tobacco, addiction to gambling and sexual activity at very early ages. In contrast, in a study undertaken in the USA [23], no significant differences were found between being tattooed and consuming alcohol or marijuana. Only in bearers of more than four tattoos was an association observed with the use of tobacco and illegal drugs. Other authors [32], who have also sought to assess the relationship between tattoos and risk behaviours, have been unable to confirm this association, arriving at the conclusion that this hypothesis is erroneous. Specifically, with respect to engaging in physical exercise, there seems to be no relationship between the intensity of such exercise and the practice of tattooing [6,29].

It is surprising that since the practice of tattooing is widespread among young people, with the possibility of adverse health effects and also a possible association with some risk behaviours, it has so far received little interest from social and health researchers, there being little scientific evidence in this regard. This situation could lead to a lack of education among young people and favour an increasing frequency of tattoos without taking into account the possible negative aspects and assuming that it is a harmless practice, which must be demonstrated.

In relation to attitudes towards tattoos, the use of the EAFT-D scale enabled us to observe that health science students’ attitudes towards tattoos are predominantly more favourable than unfavourable, although there is a high proportion of students who remain non-committal, being neither for nor against. The EAFT-D scale was previously used in a study targeted at investigating the use of tattoos among homosexual males, with the practice being perceived as something positive by part of this group [33]. In contrast, in a Brazilian university population, participants’ attitudes towards tattoos, obtained by using this same scale, proved to be predominantly negative [34]. However, another study conducted on US students using the Armstrong Tattoo Scale, which, like the EAFT-D, is a differential semantic scale designed to measure both the direction (i.e., positivity versus negativity) and intensity of attitudes, observed a positive attitude towards tattoos [13].

The limitations of this study must be taken into account. First, the monothematic nature of the survey may have led to an established response bias. In addition, since the data were self-reported by the participants, socially desirable responses may have been reported by some individuals. Moreover, the different interpretations of the terms included in the scale might possibly have interfered with the students’ true attitudes. In any future research, it would, therefore, be desirable to design and validate new instruments aimed at supplementing this information. Lastly, given that tattooing is an increasingly frequent phenomenon, conducting follow-up studies to ascertain the health consequences of tattoos in the medium-to-long term would be of the greatest interest.

## 5. Conclusions

In conclusion, health science students generally display a favourable attitude towards tattoos, although a high proportion of them are neither for nor against them. Attitudes are most positive among women, persons under 20 years of age, and nursing versus pharmacy and medical students. In terms of health habits, attitudes are more favourable among smokers, regardless of their level of physical activity, compliance with healthy eating guidelines or consumption of alcohol or other drugs.

Knowledge of attitudes towards tattooing can help build a broader knowledge base on the risk behaviours of university students and can, therefore, help plan health promotion strategies.

As far as we know, tattooing is a fairly widespread behaviour among young people, including health science students; it arouses more interest in females and the youngest students. Furthermore, the non-tattooed students frequently plan to get a tattoo, and it is commonly thought that tattooing lacks any health risks. Under the circumstances, an interest in researching this practice is needed as it has great acceptance among young people and there is a popular belief that it does not have negative health repercussions on physical or mental health. In future, only after conducting rigorous studies on the motivations for tattooing or their possible health consequences and their association with other risk behaviours would we be able to recommend or not recommend this practice with more scientific evidence.

In the future, new research should be carried out not only on the prevalence of this practice but also on its incidence and temporal evolution in the coming years. It will also be of interest to evaluate this practice in young people of different origins, educational levels and different degrees, both in health sciences and in other non-health subjects. It will be necessary to delve into the reasons for tattooing, checking how the level of knowledge of the risks involved affects this practice and the degree of satisfaction over time among tattoo bearers.

## Figures and Tables

**Table 1:** Socio-demographic characteristics of participants.

	Men	Women	Total
Socio-Demographic Characteristics	N (%)	N (%)	N (%)
Age:			
- under 20 years	44 (39.3)	103 (40.9)	147 (40.4)
- 20 years or over	68 (60.7)	149 (59.1)	217 (59.6)
Degree qualifications:			
- Medicine	37 (33.0)	77 (30.6)	114 (31.3)
- Pharmacy	58 (51.8)	116 (46.0)	174 (47.8)
- Nursing	17 (15.2)	59 (23.4)	76 (20.9)
Academic year of study:			
- 1st	49 (43.8)	111 (44.0)	160 (44.0)
- 2nd	14 (12.5)	36 (14.3)	50 (13.7)
- 3rd	12 (10.7)	21 (8.3)	33 (9.1)
- 4th	9 (8.0)	16 (6.3)	25 (6.9)
- 5th	28 (25.0)	68 (27.0)	96 (26.4)
Size of town of origin:			
- <10,000 inhabitants	16 (14.3)	71 (28.2)	87 (23.9)
- 10,000–30,000 inhabitants	18 (16.1)	32 (12.7)	50 (13.7)
- >30,000 inhabitants	77 (68.8)	139 (55.2)	216 (59.3)
- No data	1 (0.9)	10 (4.0)	11 (3.0)
Social class based on parents’ occupation:			
- I: Corporate and government administration managers (≥10 employees) and professions associated with university degrees (2nd and 3rd cycle)	21 (18.8)	28 (11.1)	49 (13.5)
- II: Corporate managers (<10 employees), professions associated with university degrees (1st cycle). Technicians, artists and sports persons	3 (2.7)	7 (2.8)	10 (2.7)
- III: Government administration employees, service workers, self-employed workers and supervisors	26 (3.2)	58 (23.0)	84 (23.1)
- IVa: Skilled manual workers	5 (4.5)	9 (3.6)	14 (3.8)
- IVb: Semi-skilled manual workers	10 (8.9)	28 (11.1)	38 (10.4)
- V: Unskilled workers	35 (31.3)	103 (40.9)	138 (37.9)
- No data	12 (10.7)	19 (7.5)	31 (8.5)
Form of coexistence:			
- Lives alone	2 (1.8)	6 (2.4)	8 (2.2)
- Lives with parents	72 (64.3)	119 (47.2)	191 (52.5)
- University residence	17 (15.2)	46 (18.3)	63 (17.3)
- Dwelling shared with other students	21 (18.8)	79 (31.3)	100 (27.5)
- No data	0 (0.0)	2 (0.8)	2 (0.5)

**Table 2:** Participants’ health habits.

Health Habits	Men	Women	Total
	N (%)	N (%)	N (%)
Level of physical activity:			
- Sedentary	26 (23.2)	74 (29.4)	100 (27.5)
- Moderate physical activity	27 (24.1)	85 (33.7)	112 (30.8)
- Intense physical activity	59 (52.7)	91 (36.1)	150 (41.2)
- No data	0 (0.0)	2 (0.8)	2 (0.5)
Healthy diet:			
- Fish (3–4 portions/week)	30 (26.8)	78 (31.0)	108 (29.7)
- Lean meat (3–4 portions/week)	62 (55.4)	133 (52.8)	195 (53.6)
- Eggs (3–4 portions/week)	30 (26.8)	49 (19.4)	79 (21.7)
- Legumes (2–4 portions/week)	79 (70.5)	169 (67.1)	248 (68.1)
- Milk, yoghurt, cheese (2–4 portions/day)	75 (67.0)	197 (78.2)	272 (74.7)
- Green leafy and other vegetables (≥2 portions/day)	25 (22.3)	77 (30.6)	102 (28)
- Fruit (≥3 portions/day)	63 (56.3)	142 (56.3)	205 (56.3)
- Bread, cereals, rice, pasta, potatoes (4–6 portions/day)	71 (63.4)	164 (65.1)	235 (64.6)
Alcohol consumption:			
- None	10 (8.9)	15 (6.0)	25 (6.9)
- Less than 280 g/week in men and <170 g/week in women	88 (78.6)	205 (81.3)	293 (80.5)
- Risk consumption (>280 g/week in men and <70 g/week in women)	8 (7.1)	22 (8.7)	30 (8.2)
- No data	6 (5.4)	10 (4.0)	16 (4.4)
Smoking:			
- Non-smoker	87 (77.7)	206 (81.7)	293 (80.5)
- Daily use	13 (11.6)	25 (9.9)	38 (10.4)
- Occasional use (<1 cigarette/day)	12 (10.7)	21 (8.3)	33 (9.1)
Use of other drugs:			
- Yes (marijuana)	12 (10.7)	5 (2.0)	17 (4.7)
- No	100 (89.3)	247 (98.0)	347 (95.3)

**Table 3:** Participants’ attitudes to tattoos.

“I Consider That Wearing Tattoos Is….”							
	1	2	3	4	5	No Data	
	N (%)	N (%)	N (%)	N (%)	N (%)	N (%)	
**Positive…**	52 (14.3)	49 (13.5)	200 (54.9)	37 (10.2)	15 (4.1)	11 (3.0)	**…Negative**
**Agreeable…**	13 (3.6)	36 (9.9)	166 (45.6)	92 (25.3)	41 (11.3)	16 (4.4)	**…Disagreeable**
**Desirable…**	39 (10.7)	93 (25.5)	154 (42.3)	43 (11.8)	20 (5.5)	15 (4.1)	**…Undesirable**
**Attractive…**	14 (3.8)	38 (10.4)	141 (38.7)	106 (29.1)	51 (14.0)	14 (3.8)	**…Ugly**
**Delicate…**	17 (4.7)	59 (16.2)	196 (53.8)	60 (16.5)	14 (3.8)	18 (4.9)	**…Aggressive**
**Right…**	17 (4.7)	51 (14.0)	195 (53.6)	63 (17.3)	22 (6.0)	16 (4.4)	**…Wrong**
**Responsible…**	34 (9.3)	62 (17.0)	194 (53.3)	45 (12.4)	12 (3.3)	17 (4.7)	**…Irresponsible**
**Appropriate…**	13 (3.6)	41 (11.3)	207 (56.9)	59 (16.2)	27 (7.4)	17 (4.7)	**…Inappropriate**
**Conformist…**	16 (4.4)	47 (12.9)	210 (57.7)	62 (17.0)	15 (4.1)	14 (3.8)	**…Rebellious**
**Conventional…**	19 (5.2)	43 (11.8)	155 (42.6)	100(27.5)	27 (7.4)	20 (5.5)	**…Unconventional**

**Table 4:** Variables shown by multiple linear regression analysis to be associated with a favourable attitude to tattoos.

Associated Variables	Coefficients (B)	95% CI	t	*p*
Constant	23.476	19.182 to 27.769	10.756	<0.001
Nursing (versus pharmacy and medical) students	1.947	0.621 to 3.273	2.888	0.004
Age (in years)	−0.183	−0.355 to −0.011	−2.095	0.037
Female gender (yes/no)	1.327	0.162 to 2.492	2.240	0.026
Smoking habit (yes/no)	1.429	0.066 to 2.793	2.063	0.040

## Data Availability

The data presented in this study are available on request from the corresponding author.
